# Commentary: False-Positive Effect in the Radin Double-Slit Experiment on Observer Consciousness as Determined With the Advanced Meta-Experimental Protocol

**DOI:** 10.3389/fpsyg.2020.00726

**Published:** 2020-04-15

**Authors:** Dean Radin, Helané Wahbeh, Leena Michel, Arnaud Delorme

**Affiliations:** Department of Research, Institute of Noetic Sciences, Petaluma, CA, United States

**Keywords:** consciousness, observer effects, quantum, experiments and analysis, double-slit

In a recent volume of this journal, Walleczek and von Stillfried (2019) critique an unpublished experiment our group conducted in 2012–2013. The study was funded by Walleczek in his role as director of a private foundation, and we did not publish the results of that experiment at Walleczek's request. The study was part of a series of experiments we and others have performed to investigate the role of the observer in quantum mechanics, an enigma discussed at length by the founders of quantum theory and recently informed by empirical evidence supporting the idea that quantum theory “should be interpreted in an observer-dependent way” (Proietti et al., 2019, p. 1; Rosenblum and Kuttner, 2006).

Walleczek and von Stillfried's (henceforth WS) primary criticism was based on what they called a false-positive result in one of eight planned comparisons. We contend that this claim is invalid because by design the experiment involved eight comparisons performed on non-overlapping data partitioned from a single dataset, and such designs require adjustment for multiple comparisons. That is, if each of eight comparisons used *p* < 0.05 as the conventional threshold for rejecting the null hypothesis, then the probability of obtaining at least one false-positive is *p* = 1 – (0.95)^8^ = 0.34, or 34%. In other words, one or more false-positives would be identified one third of the time, even in data that were pure noise. Such a high rate of false-positive “significance” provides an invalid picture of the experimental results. Despite this, WS argued that multiple-comparison adjustment was unnecessary, writing:

Since (1) neither are [sic] used multiple, or overlapping, data sets in the test of one specific null hypothesis and (2) nor are multiple null hypotheses tested using one and the same, or an overlapping, data set, calculating any type of correction for multiple comparison testing … would be in error (WS, p. 9).

Regarding that rationalization, we agree with Frane (2015): “Researchers have frequently defended their unadjusted tests explicitly on the basis that the tests were planned. The belief that stating one's hypotheses a priori eliminates or excuses Type I error inflation … has no apparent mathematical or scientific basis. Yet the myth continues to be perpetuated” (p. 6).

To obtain an independent assessment about this issue, we sought advice from a past-president of the American Statistical Association (Utts, October 2019, personal communication). She confirmed that for this experimental design correction for multiple comparisons was indeed necessary (Tukey, 1991; Curran-Everett, 2000). After applying the False Discovery Rate (FDR) algorithm to the *p*-values associated with the mean comparisons (Benjamini and Hochberg, 1995), none of the eight tests were significant. We further note that only two of the eight comparisons were predicted to show significant effects, thus by not adjusting the *p*-values, the likelihood of erroneously identifying a false-positive in the WS's design was three times greater than identifying a true-positive.

The importance that WS placed on their false-positive claim was underscored by their assertion that it “casts doubt on the scientific validity of the claimed (true)-positive effect which has been reported before ….” (WS, p. 17). That statement dismisses not only the 16 relevant experiments published by our group, but also a dozen similar studies published by three independent groups (Ibison and Jeffers, 1998; Guerrer, 2019). Eleven of those 28 experiments, all of which employed designs that did not require adjustment for multiple comparisons, were significant (at *p* < 0.05, two-tailed). Some of the 28 experiments were exploratory and as such their results should be interpreted with caution, but it is worth noting that the binomial probability of the cumulative results reported so far is *p* < 10^−7^.

Besides their invalid false-positive claim, WS repeated the terms “pre-specified” and “pre-planned” some 32 times in their article, emphasizing that the analytical methods in the experiment were established beforehand to prevent *p*-hacking. Given that emphasis, it is surprising that they do not describe those analyses. Instead, they write, “For viewing the technical details of the employed signal processing routines, this original Matlab script … can be made available upon request” (WS, p. 4). When that script is examined, it is found to include not only the mean comparisons that they focused on, but also *variance* comparisons. One of the latter comparisons, in a condition predicted to be significant, remained significant after FDR adjustment. WS do not mention this true-positive outcome, which is important because said outcome suggests an observer effect which is genuine but not predictably stable from one session to the next.

Nor do WS provide a full description of the experiment. To address that omission, in a separate article we will provide a complete account of the methods and procedures, and we will discuss additional concerns we have about claims in WS's critique.

## Author Contributions

DR wrote the initial draft. HW, LM, and AD commented on the draft. All agreed on the final version.

### Conflict of Interest

The authors declare that the research was conducted in the absence of any commercial or financial relationships that could be construed as a potential conflict of interest.
